# Developmentally sensitive neuropharmacological effects of dexamethasone in neonatal bronchopulmonary dysplasia-associated brain injury via microglial Acod1-itaconate/IL-1β signaling

**DOI:** 10.3389/fphar.2026.1840628

**Published:** 2026-07-08

**Authors:** Wen Jia, Chen Chen, Long Chen, Chan Liu, Meiyu Zhang, Jingli Yang, Yuan Shi, Li Wang

**Affiliations:** 1 Department of Pediatrics, The Second Affiliated Hospital of Chongqing Medical University, Chongqing, China; 2 Department of Neonatology, Chongqing Maternal and Child Health Hospital, Chongqing, China; 3 Department of Neonatology, Children’s Hospital of Chongqing Medical University, National Clinical Research Center for Child Health and Disorders, Ministry of Education Key Laboratory of Child Development and Disorders, Chongqing, China

**Keywords:** immunometabolism, microglia-neuron crosstalk, neuroinflammation, postnatal corticosteroids, therapeutic window

## Abstract

**Background:**

Bronchopulmonary dysplasia (BPD) in preterm infants is frequently accompanied by neurodevelopmental impairment, yet the central neuropharmacological actions of dexamethasone (DEX), a commonly used therapy for severe or evolving BPD, remain incompletely understood. In particular, whether DEX exerts timing-dependent neuroprotection in the developing brain and the mechanisms underlying such effects are unclear.

**Methods:**

We investigated the neuroprotective effects of DEX in a neonatal rat double-hit model combining prenatal maternal lipopolysaccharide exposure with postnatal hyperoxia. A tapered DEX regimen was initiated on postnatal day (P)1, P3, or P8 to evaluate the therapeutic window. Lung pathology, survival, hippocampal injury, microglial reactivity, behavioral outcomes, resting-state functional magnetic resonance imaging (rs-fMRI), targeted metabolomics, and microglia-neuron coculture experiments were used to characterize pharmacological efficacy and mechanism.

**Results:**

Among the tested regimens, DEX initiated at P3 produced the most consistent protective effects, improving alveolar structure, survival, hippocampal pathology, and microglial reactivity. P3-initiated DEX also improved recognition memory, exploratory/anxiety-related behavior, spatial memory retention, and motor coordination, and was associated with partial restoration of hippocampal functional connectivity. At the molecular level, DEX partially restored hippocampal glutamate/GABA balance, reduced Synapsin I phosphorylation, and normalized VGLUT1/VGAT associated synaptic abnormalities. Mechanistically, microglia-derived IL-1β promoted neuronal ERK/Syn1 activation, whereas DEX interrupted this inflammatory signaling axis in a microglia-neuron coculture system. Targeted metabolomics and perturbation experiments further showed that DEX increased Acod1-dependent itaconate reprogramming under inflammatory priming, thereby suppressing microglial IL-1β and downstream neuronal P-Syn1/Syn1 signaling.

**Conclusion:**

These findings identify a developmentally sensitive therapeutic window for DEX neuroprotection in neonatal BPD-associated brain injury and suggest that microglial Acod1-itaconate-dependent regulation of IL-1β/ERK/Syn1 signaling contributes to its central protective effects. This study expands the pharmacological interpretation of DEX beyond pulmonary benefit and supports an immunometabolic framework for understanding corticosteroid actions in the developing brain.

## 1 Introduction

Bronchopulmonary dysplasia (BPD) is a major complication of prematurity and is frequently accompanied by long-term neurodevelopmental impairment (Chen et al., 2023; Starke et al., 2024). Clinical and experimental studies have linked BPD to abnormalities in cognition, behavior, and brain connectivity, suggesting that neonatal lung injury and brain dysfunction are biologically coupled rather than merely co-occurring morbidities (Bell et al., 2022). However, the mechanisms linking neuroinflammation to synaptic and circuit abnormalities in BPD-associated brain injury remain incompletely understood.

Microglia are the principal innate immune cells in the developing brain and play central roles in neuroinflammatory responses, synaptic pruning, and circuit refinement. Because the preterm brain is highly vulnerable during early development, microglia are particularly sensitive to inflammatory and oxidative insults. Among the inflammatory mediators implicated in neonatal brain injury, interleukin-1β (IL-1β) is considered a major effector of synaptic dysfunction (Ansari et al., 2022; Lehtola et al., 2024). Previous studies have shown that IL-1β can activate extracellular signal-regulated kinase (ERK) signaling, whereas ERK-dependent phosphorylation of Synapsin I (Syn1) may alter synaptic vesicle dynamics and neurotransmitter release, thereby disrupting excitatory/inhibitory balance (Lee et al., 2024; Moraes et al., 2023; Yan et al., 2022). These observations raise the possibility that microglia-derived IL-1β may constitute an important mechanistic link between neuroinflammation and synaptic dysfunction.

Dexamethasone (DEX) is widely used in preterm infants with severe or evolving BPD, yet its effects on the developing brain remain controversial (Parikh and Jain, 2026; Puia-Dumitrescu et al., 2022). While early or high-dose corticosteroid exposure has been associated with adverse neurodevelopmental outcomes (Onland et al., 2023), selected clinical observations suggest that DEX may also be associated with improved neurological function under specific conditions (Chandwani et al., 2025; Jia et al., 2023). These conflicting findings suggest that the effects of DEX on the developing brain may not be uniformly beneficial or harmful, but instead may depend on developmental stage, injury context, and cellular response state (Jensen et al., 2023). Recent studies further indicate that glucocorticoids can reshape inflammatory metabolism in addition to suppressing cytokine transcription (Cummins and Goldstein, 2025). In particular, itaconate, a tricarboxylic acid cycle-derived immunometabolite generated through aconitate decarboxylase 1 (Acod1/Irg1) (Auger et al., 2024), has emerged as an important endogenous suppressor of pro-inflammatory cytokine production, including IL-1β(Gao et al., 2025; Tada and Kono, 2025). Whether DEX engages an Acod1-itaconate program in microglia during neonatal inflammatory injury, and whether this process contributes to synaptic protection, remains unknown.

Another important limitation of current preclinical studies is that many BPD models rely on hyperoxia alone and therefore do not fully recapitulate the inflammatory priming that frequently accompanies preterm neonatal injury (Huang et al., 2025; Jenkinson et al., 2023; Padam et al., 2024; Strashun et al., 2022). By contrast, the combination of inflammatory and hyperoxic insults more closely reflects the complex pathological environment of clinical BPD and may be better suited to evaluating the context-dependent central effects of DEX. In the present study, we used a neonatal double-hit model combining prenatal maternal lipopolysaccharide (LPS) exposure with postnatal hyperoxia to investigate whether the neuroprotective effects of DEX depend on postnatal treatment timing. We further tested whether these effects are associated with microglia-derived IL-1β-driven neuronal ERK/Syn1 signaling and Acod1-dependent itaconate reprogramming. By integrating histopathology, behavioral testing, resting-state functional magnetic resonance imaging (rs-fMRI), targeted metabolomics, and microglia-neuron coculture experiments, we sought to identify the treatment timing, cellular mechanism, and immunometabolic context underlying DEX-mediated neuroprotection in BPD-associated neonatal brain injury.

## 2 Materials and methods

### 2.1 Animals and experimental groups

Specific pathogen-free pregnant Sprague-Dawley rats were purchased from Weitong Lihua Experimental Animal Center (China). To establish a multifactorial neonatal injury model, dams received an intraperitoneal injection of LPS (*Escherichia coli* O55:B5, Sigma-Aldrich, L2880; 100 μg/kg in sterile saline) on embryonic day 16 (E16). Control dams received volume-matched saline. After birth, pups were exposed to either normoxia (21% O_2_) or hyperoxia (85% O_2_) from postnatal day 1 (P1) to P14. Experimental groups were defined as follows: control, prenatal saline + postnatal normoxia; BPD, prenatal LPS + postnatal hyperoxia; and BPD + DEX groups, prenatal LPS + postnatal hyperoxia with DEX initiated at P1, P3, or P8 for 7 days.

To minimize litter effects, pups from multiple litters were pooled on P1, randomly redistributed to experimental groups, and fostered by surrogate dams. Litter size was standardized to 10–12 pups per dam. To limit maternal deterioration during prolonged hyperoxia, dams caring for hyperoxia-exposed litters were rotated every 24 h with dams from normoxic cages. Male and female pups were included at an approximately 1:1 ratio. Behavioral testing, histological analyses, and image quantification were performed by investigators blinded to group allocation.

DEX sodium phosphate (MedChemExpress, HY-B1829A) was administered intraperitoneally using a tapering regimen adapted from previous neonatal steroid studies (Lee et al., 2012; Özer Bekmez et al., 2022): 0.2 mg/kg/day for 2 days, 0.1 mg/kg/day for 2 days, 0.05 mg/kg/day for 2 days, and 0.02 mg/kg/day for 1 day. Control and untreated BPD animals received volume-matched saline.

### 2.2 *In vivo* cohort design and tissue collection

Different predefined cohorts were used for longitudinal functional assessment and terminal tissue analyses. rs-fMRI and behavioral testing were performed in the same longitudinal functional cohort. Animals in this cohort underwent rs-fMRI at P35 and then behavioral testing from P42 to P51. Histological, immunofluorescence, TEM, Western blotting, ELISA, and metabolomic analyses were performed using independent tissue-collection cohorts to avoid potential confounding effects of repeated behavioral testing on molecular and histological readouts. Detailed animal allocation, mortality in the independent survival cohort, and sample use for downstream analyses are provided in Supplementary Table S1.

### 2.3 Resting-state functional magnetic resonance imaging (rs-fMRI)

Rs-fMRI was performed at P35 using a 7.0-T Bruker BioSpec scanner. Rats were anesthetized with isoflurane (3% induction, 1.5%–2% maintenance). T2-weighted echo-planar images were acquired with the following parameters: repetition time 2000 ms, echo time 9.82 ms, matrix 64 × 64, 30 slices of 1.0 mm thickness, field of view 35 × 35 mm^2^, flip angle 90°, and 300 volumes.

Data were processed in SPM12, including slice-timing correction, motion correction, exclusion of volumes with framewise displacement >0.3 mm, spatial normalization to the Paxinos rat brain atlas, and temporal band-pass filtering (0.01–0.1 Hz). Seed-based connectivity analysis was performed using bilateral hippocampal regions of interest. Correlation coefficients were Fisher Z-transformed before group analysis. Statistical significance was assessed using Gaussian random field correction with a voxel-level threshold of P < 0.001 and a cluster-level threshold of P < 0.05, with a minimum cluster size of 100 voxels.

### 2.4 Behavioral testing

Behavioral testing was performed from P42 to P51 in the same animals that had undergone rs-fMRI at P35. All tests were conducted during the light phase in a quiet behavioral testing room under controlled environmental conditions, including a temperature of 22 °C–24 °C, 40%–60% humidity, and consistent illumination. Investigators performing the behavioral tests and data analysis were blinded to group allocation. Before each test, rats were transferred to the behavioral testing room and allowed to acclimate for at least 30 min. To minimize carry-over effects related to exploratory or memory-dependent tasks, no more than one major behavioral paradigm was performed on the same day, except for habituation or training sessions required within the same task. Apparatuses and objects were cleaned with 70% ethanol between animals.

The behavioral sequence was as follows: open field test, novel object recognition, Morris water maze, and rotarod test. The open field test was performed first to avoid interference from prior spatial learning or motor training. Rats were allowed to explore the arena for 5 min, and total distance traveled and time spent in the center zone were analyzed using ANY-maze software. For novel object recognition, rats were familiarized with two identical objects for 10 min, and 24 h later one object was replaced with a novel object for the test session. The discrimination index was calculated to evaluate recognition memory.

In the Morris water maze, rats underwent four trials per day for five consecutive days. Escape latency to locate the hidden platform was recorded during the acquisition phase. On day 6, the platform was removed, and the percentage of time spent in the target quadrant was measured during the probe trial. Water-maze trajectories and parameters were analyzed using ANY-maze software. Finally, motor coordination was assessed using the rotarod test. Rats were placed on an accelerating rotarod from 4 to 40 rpm over 300 s, and latency to fall was recorded.

### 2.5 Time points and tissue collection for *in vivo* analyses

Lung tissues were collected at P15 and P28 for histological evaluation of alveolar development. For lung histology, the lungs were inflated and fixed with 4% paraformaldehyde under standardized pressure, embedded in paraffin, and cut into 5-μm transverse sections for hematoxylin and eosin staining. P15 was selected to assess lung injury immediately after completion of postnatal hyperoxic exposure, whereas P28 was used to evaluate persistent or recovery-stage pulmonary changes. Body weight and brain weight were measured at P28. Survival was monitored daily from birth to P28.

Hippocampal tissues for early brain histology, Iba1 immunofluorescence, and ultrastructural analysis were collected at P15, immediately after completion of hyperoxic exposure. Rats were deeply anesthetized with an overdose of pentobarbital sodium before tissue collection. For histological and immunofluorescence analyses, animals were transcardially perfused with ice-cold PBS followed by 4% paraformaldehyde. Brains were removed, post-fixed in 4% paraformaldehyde, cryoprotected in graded sucrose solutions, and cut into 20-μm coronal cryosections. Hippocampal sections containing the dentate gyrus and CA1 regions were selected for Nissl staining and immunofluorescence analysis. For transmission electron microscopy, small hippocampal tissue blocks were rapidly dissected from comparable regions and fixed in 2.5% glutaraldehyde for ultrastructural processing. Ultrathin sections of 60–80 nm were prepared using a Leica UC7 ultramicrotome.

Terminal hippocampal tissues for neurotransmitter profiling and molecular assays were collected at P52 from the terminal molecular cohort.

### 2.6 Body and brain weight measurement

Body weight was recorded at P28 before euthanasia. After euthanasia and brain removal, whole brain weight was measured immediately using an electronic balance. Brain weight was analyzed as absolute brain weight, and body weight was recorded in parallel to assess systemic growth.

### 2.7 Primary hippocampal neuron culture

Primary hippocampal neurons were isolated from E18 rat embryos of both sexes without sex-based separation. Hippocampi were dissected in ice-cold Hank’s balanced salt solution, digested in 0.25% trypsin-EDTA (Gibco, #25200056) containing DNase I (10 μg/mL; Sigma-Aldrich, #DN25) for 15 min at 37 °C, and the digestion was stopped with DMEM containing 10% fetal bovine serum. Tissue was mechanically dissociated, filtered through a 70-μm cell strainer, centrifuged at 200 *g* for 5 min, and resuspended in Neurobasal-A medium supplemented with 2% B-27, 0.5 mM GlutaMAX, and 1% penicillin-streptomycin.

Cells were plated at 2 × 10^5^ cells/cm^2^ on poly-D-lysine- and laminin-coated culture surfaces and maintained at 37 °C in 5% CO_2_. Half of the medium was replaced every 48 h. Neurons were used at 7–10 days *in vitro*. Neuronal purity (>95%) was confirmed by MAP2 immunostaining.

### 2.8 Primary microglia isolation and culture

Primary microglia were prepared from P1–P3 rat pups of both sexes. Cortical tissue was dissociated using 0.25% trypsin-EDTA and DNase I and plated in T75 flasks in DMEM/F-12 supplemented with 10% fetal bovine serum and 1% penicillin-streptomycin. Mixed glial cultures were maintained for 10–14 days, and microglia were collected by orbital shaking (200 rpm, 2 h, 37 °C). Microglial purity (>95%) was confirmed by Iba1 immunostaining.

### 2.9 Microglia-neuron transwell coculture and treatment sequence

Primary hippocampal neurons were isolated from E18 rat embryos and cultured in the lower chamber. Primary microglia were prepared from P1–P3 rat pups and seeded into the upper chamber at 5 × 10^4^ cells/cm^2^. Coculture experiments were performed using a 1:1 mixture of neuronal and microglial media supplemented with 1% fetal bovine serum. This reduced-serum condition was selected to maintain neuronal compatibility while supporting short-term microglial viability.

For genetic perturbation experiments, microglia were transduced with si-Acod1, si-IL-1β, or OE-IL-1β constructs for 4 h. After replacement with complete medium, cells were maintained for 48 h before being used for coculture experiments. Target knockdown or overexpression was confirmed by qPCR or Western blotting before downstream analyses.

After assembly of the Transwell coculture system, 4-octyl-itaconate (4-OI, 100 μM) was added to the microglial compartment 2 h before inflammatory stimulation where indicated. In ERK modulation experiments, Trametinib or EGF was added to the neuronal compartment before inflammatory stimulation and maintained throughout the coculture period. The coculture system was then treated with LPS (100 ng/mL) and/or DEX (100 nM) for 12 h. For hyperoxic exposure, coculture plates were placed in a humidified three-gas incubator containing 85% O_2_/5% CO_2_ for 12 h; control cultures were maintained under normoxia. At the end of coculture, microglia from the upper chamber and hippocampal neurons from the lower chamber were harvested separately. Microglial Acod1 and IL-1β, and neuronal P-ERK/ERK and P-Syn1/Syn1, were analyzed by Western blotting.

Primary microglia and hippocampal neurons were prepared from independent litters. Each *in vitro* experiment was repeated using at least three independent biological culture preparations. For each biological preparation, two to three technical wells were included per condition, and technical replicates were averaged before statistical analysis. The independent biological preparation, rather than individual wells, was used as the statistical unit.

### 2.10 *In vitro* experimental groups

The *in vitro* coculture experiments were organized according to the specific mechanistic question being tested. For the core inflammatory coculture experiments, the groups were Control, DEX, O_2_+LPS, and O_2_+LPS + DEX. In this context, O_2_+LPS refers to combined hyperoxic and inflammatory stimulation in the coculture system, with exposure to 85% O_2_ and LPS treatment, and does not denote a separate *in vivo* animal group.

For IL-1β perturbation experiments, additional groups included O_2_+LPS + DEX + OE-IL-1β and O_2_+LPS + si-IL-1β. For neuronal ERK pathway modulation, the groups included Control, O_2_+LPS, O_2_+LPS + Trametinib, and O_2_+LPS + EGF. For Acod1-itaconate pathway experiments, the groups included O_2_+LPS, O_2_+LPS + DEX, O_2_+LPS + DEX + si-Acod1, and O_2_+LPS + DEX + si-Acod1+4-OI, as indicated in the corresponding figure legends.

For microglial metabolomic profiling, primary microglia were analyzed under normoxia, DEX alone, O_2_, O_2_+DEX, LPS, and LPS + DEX conditions to compare hyperoxic and inflammatory priming contexts.

### 2.11 Western blotting

Hippocampal tissues, lung tissues, primary microglia, or primary hippocampal neurons were lysed in ice-cold RIPA buffer supplemented with protease and phosphatase inhibitor cocktails. Lysates were centrifuged at 12,000 × g for 15 min at 4 °C, and supernatants were collected. Protein concentrations were determined using a BCA protein assay kit. Equal amounts of protein, usually 30 μg per lane, were separated on 4%–20% gradient SDS-PAGE gels and transferred onto PVDF membranes.

Membranes were blocked with rapid protein blocking buffer for Western blotting (Beyotime Biotechnology, China) for 15 min at room temperature according to the manufacturer’s instructions. After blocking, membranes were incubated overnight at 4 °C with the indicated primary antibodies. Membranes were then washed three times with TBST and incubated with HRP-conjugated anti-rabbit or anti-mouse secondary antibodies for 1 h at room temperature. Protein bands were visualized using enhanced chemiluminescence and imaged with a ChemiDoc MP imaging system.

Band intensities were quantified using ImageJ. Total protein expression was normalized to β-actin, α-tubulin, GAPDH, or Tubulin as indicated in each figure. Phosphorylated proteins were normalized to their corresponding total proteins, including P-ERK/ERK and P-Syn1/Syn1. For coculture experiments, microglia and neurons were harvested separately: Acod1 and IL-1β were measured in microglial lysates, whereas P-ERK/ERK and P-Syn1/Syn1 were measured in neuronal lysates.

The relevant antibody information is provided in Supplementary Table S2. Uncropped Western blot images are provided in Supplementary Figure S3.

### 2.12 Enzyme-linked immunosorbent assay

Lung tissues were homogenized in ice-cold lysis buffer containing protease inhibitors and centrifuged at 12,000 × g for 15 min at 4 °C. Supernatants were collected for ELISA (R&D Systems: VEGFA #RRV01, MMP9 #RMP900). MMP9 and VEGFA concentrations were measured using commercially available ELISA kits according to the manufacturer’s instructions. Absorbance was measured at 450 nm with wavelength correction at 540 nm. Concentrations were normalized to total protein and expressed as pg/mg protein.

### 2.13 Targeted metabolomics

Targeted metabolomic profiling was performed on two types of samples. For *in vivo* neurotransmitter analysis, hippocampal tissues were collected at P52 from an age-matched terminal molecular cohort that was not subjected to behavioral testing. Tissues were rapidly dissected on ice, snap-frozen in liquid nitrogen, and stored at −80 °C until analysis. These samples were used to quantify neurotransmitter-related metabolites, including L-glutamic acid and γ-aminobutyric acid. For *in vitro* metabolic analysis, primary microglial cell pellets were collected after the indicated stimulation conditions and used to quantify TCA cycle-related metabolites and itaconate.

Metabolomic profiling was performed using an ExionLC AD system coupled to a QTRAP 6500+ mass spectrometer. Metabolites were separated on a C18 reverse-phase column maintained at 35 °C using 0.1% formic acid in water as mobile phase A and 0.1% formic acid in acetonitrile as mobile phase B at a flow rate of 0.3 mL/min. Detection was performed in multiple reaction monitoring mode with electrospray ionization.

To minimize batch effects, samples from different experimental groups were randomized before LC–MS/MS injection. Pooled quality-control samples were prepared by mixing equal aliquots from all study samples and were injected after every 10 experimental samples to monitor instrument stability. The predefined acceptance criteria required retention time stability and peak area relative standard deviation < 15% in QC samples. Sample inclusion or exclusion based on these QC criteria was performed before group decoding. The experimenters responsible for peak integration, normalization, and QC evaluation were blinded to group allocation.

Raw data were processed using SCIEX OS and quantified using internal standard calibration. Metabolite annotation was supported by matching retention times and fragmentation patterns to authentic standards or an in-house metabolite library. For metabolomics data, P values were adjusted for multiple testing using the Benjamini–Hochberg false discovery rate procedure.

### 2.14 Immunofluorescence/immunocytochemistry

For tissue immunofluorescence, coronal brain sections containing the hippocampus were selected for analysis. P15 hippocampal sections were used to assess early microglial reactivity by Iba1 staining in the dentate gyrus and CA1 regions. P52 hippocampal sections were used for synaptic marker staining, including VGLUT1, VGAT, and phospho-Synapsin I. Sections were permeabilized with 0.1% Triton X-100, blocked with 5% bovine serum albumin, and incubated overnight at 4 °C with the indicated primary antibodies. After washing, sections were incubated with species-appropriate Alexa Fluor-conjugated secondary antibodies for 1 h at room temperature. Nuclei were counterstained with DAPI.

For immunocytochemistry, primary hippocampal neurons and primary microglia grown on coverslips were fixed with 4% paraformaldehyde for 15 min, permeabilized with 0.1% Triton X-100, blocked with 5% bovine serum albumin for 1 h, and incubated overnight at 4 °C with antibodies against MAP2 or Iba1, respectively. After washing, cells were incubated with species-appropriate Alexa Fluor-conjugated secondary antibodies for 1 h at room temperature, and nuclei were counterstained with DAPI. Cell purity was calculated as MAP2^+^ or Iba1^+^ cells divided by total DAPI^+^ cells × 100%.

Images were acquired using an Olympus SpinSR spinning-disk confocal microscope with ×20 and ×40 objectives. Laser power, exposure time, gain, and offset settings were kept identical within each staining batch. For each animal, three coronal sections and three non-overlapping fields per hippocampal region were analyzed. Iba1-positive cells and DAPI-positive nuclei were counted within predefined DG and CA1 regions. The Iba1^+^ cell proportion was calculated as Iba1^+^ cells divided by total DAPI^+^ cells and normalized to the mean value of the Control group. For synaptic marker analysis, mean fluorescence intensity or puncta-related measurements were quantified in predefined hippocampal regions using the same threshold settings across groups. The animal was used as the statistical unit. Quantification was performed by investigators blinded to group allocation.

Detailed information on the primary and secondary antibodies used for immunofluorescence and immunocytochemistry is provided in Supplementary Table S2.

### 2.15 Histology and ultrastructural analysis

For Nissl staining, 20-μm coronal cryosections containing the P15 hippocampus were processed using a commercial Nissl staining kit according to the manufacturer’s instructions. Neurons with clear nuclei and preserved cytoplasmic morphology were considered structurally intact.

For lung histology, P15 and P28 lung sections were stained with hematoxylin and eosin. Five randomly selected non-overlapping fields per section were imaged at ×200 magnification. Mean linear intercept and radial alveolar count were quantified using ImageJ according to standard criteria (Dunnill, 1962). The animal, rather than individual fields, was used as the statistical unit.

P15 hippocampal tissue blocks were fixed in 2.5% glutaraldehyde, post-fixed in 1% osmium tetroxide, dehydrated through graded ethanol and acetone, embedded in SPI 812 resin, and cut into 60–80 nm ultrathin sections using a Leica UC7 ultramicrotome. Sections were stained with uranyl acetate and lead citrate and examined using a JEM 1400 transmission electron microscope. Representative images were obtained from comparable hippocampal regions across groups, and analysis was performed by investigators blinded to group allocation.

### 2.16 Statistical analysis

Data are presented as mean ± SEM unless otherwise stated. Normality was assessed using the Shapiro–Wilk test. For two-group comparisons, normally distributed data were analyzed using unpaired Student’s t-test, whereas non-normally distributed data were analyzed using the Mann–Whitney U test. For non-factorial comparisons among three or more groups, normally distributed data were analyzed using one-way ANOVA followed by Tukey’s multiple-comparisons test, whereas non-normally distributed data were analyzed using the Kruskal–Wallis test followed by Dunn’s multiple-comparisons test.

For experiments involving two independent factors, such as inflammatory/hyperoxic stimulation and DEX treatment, two-way ANOVA was used followed by Sidak’s or Tukey’s multiple-comparisons test, as appropriate. Morris water maze acquisition data were analyzed using repeated-measures two-way ANOVA with group and training day as factors. Survival curves were analyzed using the log-rank test.

For histological and immunofluorescence analyses, values from multiple fields or sections were averaged for each animal, and the animal was used as the statistical unit. For *in vitro* experiments, independent biological culture preparations were used as the statistical unit, and technical replicates were averaged before statistical analysis. Exact n values and statistical tests are provided in the corresponding figure legends. *P* < 0.05 was considered statistically significant.

### 2.17 Study approval

All animal procedures were approved by the Institutional Animal Care and Use Committee of Chongqing Medical University (Approval No. IACUC20240412005) and were conducted in accordance with the Guide for the Care and Use of Laboratory Animals.

## 3 Results

### 3.1 Among the tested postnatal initiation times, P3 showed the most consistent protective effect of DEX in the neonatal double-hit BPD model

**FIGURE 1:**
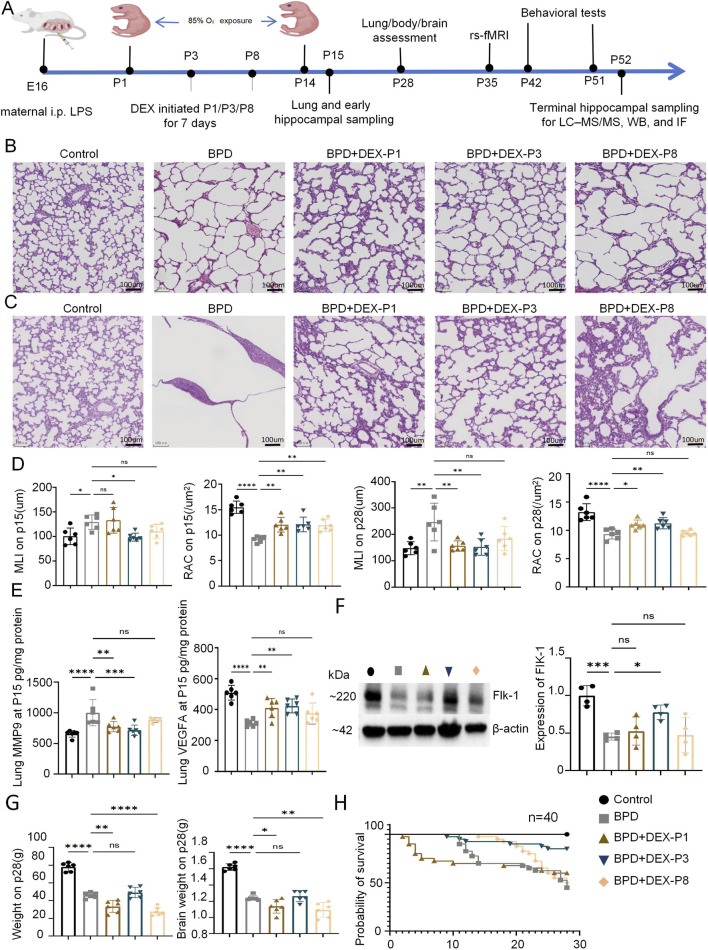
Postnatal timing determines the pulmonary efficacy and survival benefit of DEX in a neonatal double-hit BPD model. **(A)** Experimental timeline. Pregnant rats received an intraperitoneal injection of LPS at embryonic day 16 (E16). Neonatal rats were exposed to 85% O_2_ from postnatal day 1 (P1) to P14. DEX treatment was initiated at P1, P3, or P8 and continued for 7 days using a tapering regimen. Lung tissues and early hippocampal tissues were collected at P15 for pulmonary histology, early brain histology, microglial reactivity assessment, and ultrastructural analysis. Lung/body/brain assessments were performed at P28. Resting-state functional MRI was performed at P35, and behavioral tests were conducted from P42 to P51. Terminal hippocampal tissues were collected at P52 for neurotransmitter profiling and molecular analyses, including LC–MS/MS, Western blotting, and immunofluorescence. **(B,C)** Representative H&E-stained lung sections at P15 and P28, respectively. Scale bars, 100 μm. **(D)** Quantification of mean linear intercept (MLI) and radial alveolar count (RAC) at P15 and P28, n = 6 animals per group, with five random fields analyzed per animal. **(E)** Pulmonary MMP9 and VEGFA levels at P15 measured by ELISA (n = 6). **(F)** Representative Western blot and quantification of pulmonary Flk-1/VEGFR2 expression (n = 4). **(G)** Body weight and brain weight at P28 (n = 6). **(H)** Kaplan–Meier survival curves through P28. Survival analysis was performed in an independent survival cohort with 40 pups initially included in each group. Deaths before P28 were recorded as events, and animals alive at P28 were censored at the end of follow-up. Planned euthanasia for tissue collection was performed in separate cohorts and was not included in the survival analysis. Survival curves were compared using the log-rank test. Data are presented as mean ± SEM. Multiple-group comparisons were analyzed using one-way ANOVA followed by Tukey’s *post hoc* test or the Kruskal–Wallis test followed by Dunn’s multiple-comparisons test, as appropriate. *P < 0.05, **P < 0.01, ***P < 0.001, ****P < 0.0001; ns, not significant.

To better recapitulate the multifactorial pathogenesis of BPD-associated brain injury, we established a neonatal double-hit model by combining prenatal maternal LPS exposure with postnatal hyperoxia, as illustrated in the experimental timeline (Figure 1A). We next evaluated whether the timing of DEX administration influenced treatment efficacy by initiating DEX at P1, P3, or P8. Histological analysis of the lung revealed marked alveolar simplification and septal thickening in the BPD group, consistent with impaired alveolar development. Among the three treatment schedules, DEX initiated at P3 produced the most favorable overall profile in lung architecture at both P15 and P28, whereas treatment initiated at P1 or P8 conferred limited or no clear benefit (Figures 1B,C).

Morphometric analysis further confirmed these observations. The BPD group showed increased MLI and reduced RAC, indicating disrupted alveolarization. P3-initiated DEX partially normalized these indices, whereas the P1 and P8 regimens were less effective (Figure 1D). Consistent with the histological findings, pulmonary MMP9 and VEGFA levels, together with Flk1/VEGFR2 expression, were altered in BPD animals and were partially restored by DEX treatment, with the P3 schedule again showing the most favorable profile (Figures 1E,F).

Because systemic steroid treatment may affect somatic and brain growth, body and brain weights were assessed at P28. BPD animals showed reduced body and brain weights compared with controls. P3-initiated DEX did not significantly restore these growth-related indices compared with the BPD group, but it did not further aggravate body or brain weight reduction. By contrast, DEX initiated at P1 or P8 showed limited benefit and appeared less favorable in terms of growth-related outcomes (Figure 1G). Therefore, the P3 regimen showed the most favorable overall balance among the tested schedules when lung structure, survival, and growth-related safety were considered together. Survival analysis further showed that DEX administered at P3 provided the clearest improvement in survival through P28 (Figure 1H), indicating that DEX efficacy depends on the timing of postnatal initiation in this neonatal double-hit model.

Collectively, these results identify P3 as the most favorable initiation time among the schedules tested and provide the basis for subsequent mechanistic analyses focused on this regimen.

### 3.2 P3-initiated DEX attenuates hippocampal injury and microglial reactivity in the double-hit neonatal model

**FIGURE 2:**
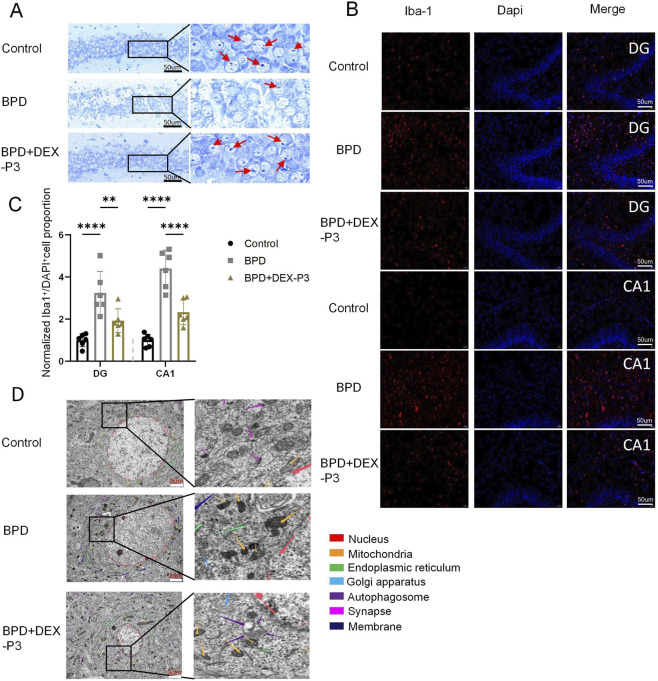
P3-initiated DEX attenuates hippocampal injury and microglial reactivity in the double-hit neonatal model. Hippocampal tissues were collected at P15, immediately after completion of hyperoxic exposure, for Nissl staining, Iba1 immunofluorescence, and TEM analysis. **(A)** Representative Nissl staining of the hippocampal formation. Arrows indicate Nissl bodies, reflecting neuronal structural integrity. Scale bar, 50 μm. **(B)** Representative immunofluorescence images showing Iba1-positive microglia in the dentate gyrus (DG) and CA1 regions of the hippocampus. Nuclei were counterstained with DAPI. Scale bar, 50 μm. **(C)** Quantification of normalized Iba1^+^/DAPI^+^ cell proportion in the DG and CA1 regions. Iba1^+^ cell proportion was calculated as Iba1^+^ cells divided by total DAPI^+^ cells and normalized to the mean value of the Control group. **(D)** Transmission electron microscopy (TEM) images of hippocampal ultrastructure. The BPD group exhibited mitochondrial shrinkage and increased electron density. These alterations were attenuated in the BPD + DEX-P3 group. Colored annotations indicate representative subcellular structures, including nucleus, mitochondria, endoplasmic reticulum, Golgi apparatus, autophagosome, synapse, and membrane. Scale bar, 2 μm. N = 6 animals per group; three sections per animal and three non-overlapping fields per hippocampal region were analyzed. Values from multiple fields were averaged for each animal before statistical analysis, and the animal was used as the statistical unit. Quantification was performed by investigators blinded to group allocation. Dashed vertical lines separate different brain regions; statistical comparisons were performed within each region, not between DG and CA1 regions. Data are presented as mean ± SEM. Statistical analysis was performed using one-way ANOVA followed by Tukey’s multiple-comparisons test for comparisons among Control, BPD, and BPD + DEX-P3 groups. *P < 0.05, **P < 0.01, ***P < 0.001, ****P < 0.0001.

We next examined whether the double-hit insult induced pathological changes in the hippocampus and whether these changes were modified by DEX treatment initiated at P3. Nissl staining demonstrated clear neuronal structural disruption in the BPD hippocampus, with reduced preservation of Nissl bodies compared with controls. In contrast, the BPD + DEX-P3 group exhibited improved neuronal morphology and greater preservation of Nissl bodies, indicating attenuation of hippocampal injury (Figure 2A).

Given the central role of microglia in neuroinflammatory injury, we assessed microglial reactivity by Iba1 immunofluorescence in the dentate gyrus (DG) and CA1 regions. Iba1 immunofluorescence revealed enhanced microglial reactivity in the DG and CA1 regions of BPD rats (Figure 2B). Quantitative analysis showed that the normalized proportion of Iba1^+^ cells among DAPI^+^ cells was significantly increased in the BPD group compared with controls, whereas this increase was attenuated in the BPD + DEX-P3 group (Figure 2C).

Ultrastructural analysis by transmission electron microscopy further revealed severe subcellular injury in the BPD hippocampus, including mitochondrial shrinkage, increased electron density, and altered autophagosome-like structures. These abnormalities were attenuated in the BPD + DEX-P3 group (Figure 2D).

Together, the histological, immunofluorescence, and ultrastructural findings indicate that the double-hit paradigm induces hippocampal injury accompanied by prominent microglial reactivity, and that DEX initiated at P3 attenuates these pathological changes.

### 3.3 P3-initiated DEX improves behavioral performance and partially restores hippocampal functional connectivity

**FIGURE 3:**
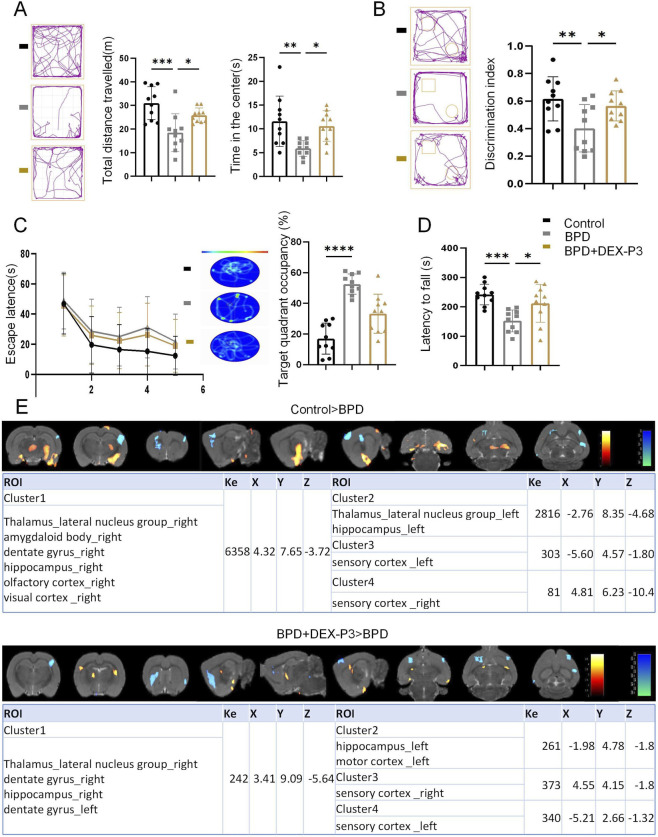
P3-initiated DEX improves behavioral performance and partially restores hippocampal functional connectivity in the double-hit neonatal model. **(A)** Open field test. Left, total distance traveled; right, time spent in the center zone. **(B)** Novel object recognition (NOR) test. The discrimination index is shown as a measure of recognition memory. **(C)** In the Morris water maze, escape latency during the 5-day acquisition phase was analyzed using repeated-measures two-way ANOVA. No significant group difference was detected during acquisition. Spatial memory retention was assessed in the probe trial by measuring the percentage of time spent in the target quadrant. **(D)** Rotarod performance, presented as latency to fall. **(E)** Resting-state functional magnetic resonance imaging (rs-fMRI) showing hippocampal functional connectivity. Upper panel, regions with reduced hippocampal connectivity in BPD versus control animals. Lower panel, regions showing increased hippocampal connectivity in BPD animals treated with DEX at P3. Cluster size (Ke) and peak stereotaxic coordinates are indicated in the tables. Statistical parametric maps were thresholded at P < 0.05 with GRF correction. These data show that P3-initiated DEX treatment improves hippocampus-related behavioral outcomes and was associated with partial restoration of hippocampal functional connectivity in the double-hit insult. N = 10 animals per group. Data are presented as mean ± SEM. NOR, open-field, probe trial, and rotarod data were analyzed by one-way ANOVA followed by Tukey’s multiple-comparisons test; escape latency across acquisition days was analyzed by two-way repeated-measures ANOVA. *P < 0.05, **P < 0.01, ***P < 0.001, ****P < 0.0001.

To determine whether the structural protection conferred by DEX translated into functional benefit, we performed a battery of behavioral tests. In the open field test, BPD animals showed increased locomotor activity together with reduced time spent in the center zone, consistent with altered exploratory/anxiety-related behavior. These abnormalities were partially corrected by DEX treatment at P3 (Figure 3A). In the novel object recognition task, BPD rats exhibited a reduced discrimination index, indicating impaired recognition memory, whereas P3-initiated DEX significantly improved performance (Figure 3B).

In the Morris water maze, escape latency decreased across the 5-day acquisition phase in all groups, and no significant group difference was detected during acquisition. In the probe trial, BPD rats spent significantly less time in the target quadrant, indicating impaired spatial memory retention, and this deficit was partially improved by P3-initiated DEX (Figure 3C). Motor coordination was also compromised in BPD animals, with reduced latency to fall on the rotarod, and this defect was improved after DEX treatment (Figure 3D).

We next examined whether these behavioral improvements were accompanied by partial recovery of hippocampal functional connectivity. rs-fMRI showed that the BPD group exhibited reduced hippocampal functional connectivity with multiple regions, including the thalamus, dentate gyrus, hippocampal subregions, and sensorimotor cortex. Notably, DEX initiated at P3 partially restored hippocampal connectivity with these regions (Figure 3E). These findings indicate that P3-initiated DEX improves hippocampus-related behavioral outcomes and is associated with partial restoration of hippocampal functional connectivity disrupted by the double-hit neonatal insult.

### 3.4 DEX restores hippocampal neurotransmitter balance and Syn1-associated synaptic marker abnormalities

**FIGURE 4:**
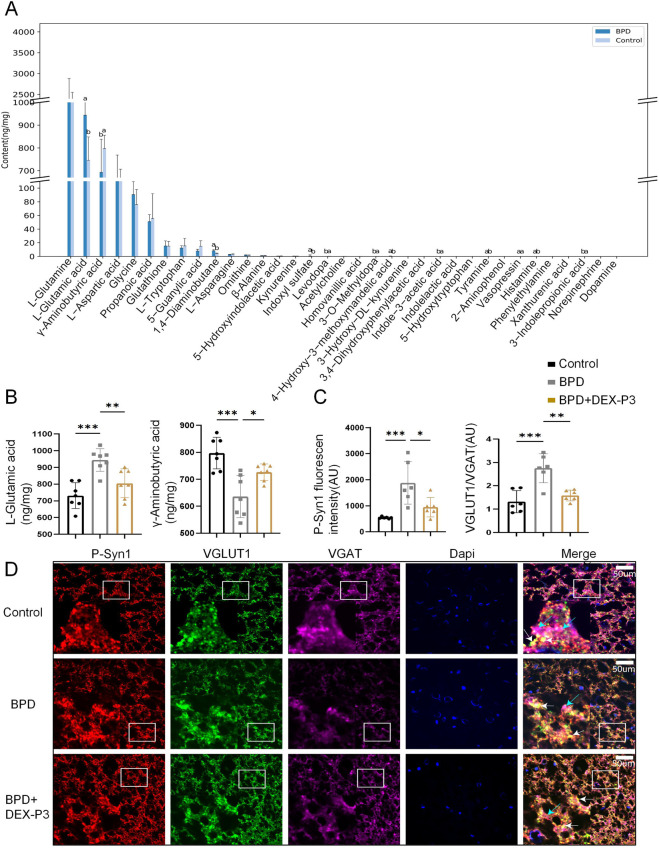
DEX restores hippocampal neurotransmitter balance and Syn1-associated synaptic marker abnormalities in BPD rats. Hippocampal tissues for neurotransmitter profiling were collected at P52 from the terminal molecular cohort. Samples were randomized before LC–MS/MS injection, and pooled QC samples were injected after every 10 experimental samples. **(A)** Targeted LC–MS/MS profiling of hippocampal neurotransmitters in control and BPD rats. **(B)** Quantification of L-glutamic acid (Glu) and γ-aminobutyric acid (GABA) concentrations, showing an excitatory/inhibitory imbalance in the BPD hippocampus and partial normalization after DEX treatment initiated at P3 (n = 7). **(C)** Quantification of P-Syn1 fluorescence intensity and VGLUT1/VGAT ratio (n = 6). **(D)** Representative immunofluorescence images of P-Syn1, VGLUT1, and VGAT in the hippocampus. White arrows indicate overlapping P-Syn1 and VGLUT1 signals; cyan arrows indicate overlapping P-Syn1 and VGAT signals. Scale bar, 50 μm. Data are presented as mean ± SEM. *P < 0.05, **P < 0.01, ***P < 0.001, ****P < 0.0001.

To investigate the molecular basis underlying the behavioral and circuit-level recovery, we performed targeted LC–MS/MS profiling of hippocampal neurotransmitters. Compared with controls, the BPD hippocampus exhibited a clear shift in neurotransmitter homeostasis, characterized by increased L-glutamic acid and decreased γ-aminobutyric acid (GABA), consistent with excitatory/inhibitory imbalance (Figures 4A,B). P3-initiated DEX partially normalized both glutamate and GABA levels, indicating restoration of hippocampal neurotransmitter balance.

We then examined synaptic markers related to vesicular neurotransmitter handling and Synapsin I phosphorylation. Quantitative analysis confirmed increased P-Syn1 fluorescence intensity and an elevated VGLUT1/VGAT ratio in BPD animals, both of which were reduced by DEX treatment (Figure 4C). Representative immunofluorescence images are shown in Figure 4D.

These data indicate that the double-hit insult disrupts hippocampal neurotransmitter homeostasis and Syn1-associated synaptic marker profiles, whereas P3-initiated DEX partially restores excitatory/inhibitory balance at both the neurochemical and synaptic levels.

### 3.5 Microglial IL-1β drives neuronal ERK/Syn1 signaling and is suppressed by DEX

**FIGURE 5:**
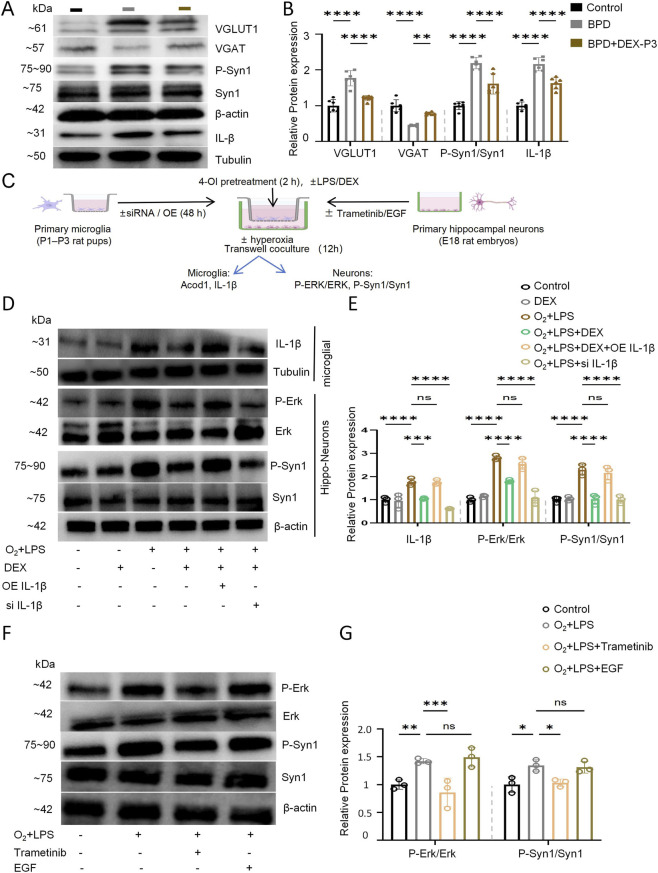
Microglial IL-1β regulates neuronal ERK/Syn1 signaling and is suppressed by DEX. **(A)** Representative immunoblots of hippocampal lysates from control, BPD, and BPD + DEX-P3 rats showing VGLUT1, VGAT, P-Syn1, Syn1, IL-1β, β-actin, and Tubulin. **(B)** Densitometric quantification of VGLUT1, VGAT, P-Syn1/Syn1, and IL-1β in hippocampal lysates. P3-initiated DEX reduced IL-1β expression and Syn1 phosphorylation and partially restored the VGLUT1/VGAT balance *in vivo* (n = 6). **(C)** Schematic workflow of the primary microglia-neuron Transwell coculture system. Primary microglia were isolated from P1–P3 rat pups and seeded in the upper chamber, whereas primary hippocampal neurons were isolated from E18 rat embryos and cultured in the lower chamber. Where indicated, microglia were subjected to siRNA or overexpression manipulation before coculture and used 48 h later. 4-OI was added 2 h before LPS/DEX stimulation. LPS and/or DEX treatment was applied for 12 h, and hyperoxia was applied to the coculture system during the 12-h stimulation period. In selected experiments, neurons were pretreated with Trametinib or EGF to modulate ERK signaling. Microglia and neurons were harvested separately for downstream analyses. **(D)** Representative immunoblots of IL-1β in microglia and P-ERK, ERK, P-Syn1, and Syn1 in hippocampal neurons under the indicated coculture conditions. DEX reduced microglial IL-1β expression and neuronal ERK/Syn1 activation under O_2_+LPS stimulation. Overexpression of IL-1β in microglia attenuated the inhibitory effect of DEX, whereas knockdown of IL-1β enhanced the suppression of neuronal ERK/Syn1 signaling. **(E)** Densitometric quantification of IL-1β in microglia and P-ERK/ERK and P-Syn1/Syn1 in hippocampal neurons under the conditions shown in **(D)** (n = 3). **(F)** Representative immunoblots showing pharmacological modulation of neuronal ERK signaling in the inflammatory coculture system. Hippocampal neurons were treated with Trametinib, an ERK pathway inhibitor, or EGF, an ERK pathway activator, followed by detection of P-ERK, ERK, P-Syn1, and Syn1 **(G)**. Densitometric quantification of the immunoblots shown in **(F)**, demonstrating that ERK inhibition by Trametinib decreased, whereas ERK activation by EGF increased, P-ERK/ERK and P-Syn1/Syn1 levels (n = 3). Together, these findings support a model in which microglia-derived IL-1β promotes neuronal ERK-dependent Syn1 phosphorylation, and DEX interrupts this inflammatory signaling axis. Dashed vertical lines separate different targets; statistical comparisons were performed within each target, not between targets. Data are presented as mean ± SEM from independent experiments as indicated in the figure. For the four core coculture groups, two-way ANOVA was used to assess the effects of O_2_+LPS stimulation, DEX treatment, and their interaction. Genetic perturbation and ERK modulation experiments were analyzed using one-way ANOVA with prespecified *post hoc* comparisons. *P < 0.05, **P < 0.01, ***P < 0.001, ****P < 0.0001.

To determine whether inflammatory signaling contributed to the synaptic abnormalities observed *in vivo*, we first examined hippocampal lysates from control, BPD, and BPD + DEX-P3 rats. Immunoblot analysis showed increased VGLUT1, elevated P-Syn1/Syn1, and increased IL-1β, together with reduced VGAT expression, in the BPD group. These changes were partially reversed by DEX treatment initiated at P3 (Figures 5A,B), suggesting that DEX suppresses hippocampal inflammatory signaling while restoring synaptic homeostasis *in vivo*.

We next established a Transwell coculture system to assess microglia-to-neuron signaling, with primary microglia placed in the upper chamber and primary hippocampal neurons in the lower chamber (Figure 5C). The purity of the primary cultures was verified by MAP2 staining for neurons and Iba1 staining for microglia (Supplementary Figure S1). Under O_2_+LPS stimulation, DEX markedly reduced IL-1β expression in microglia and simultaneously decreased neuronal P-ERK/ERK and P-Syn1/Syn1, indicating that the anti-inflammatory effect of DEX on microglia was associated with suppression of downstream neuronal signaling (Figures 5D,E).

To test whether microglial IL-1β was functionally required for this effect, we manipulated IL-1β expression in microglia. Overexpression of IL-1β attenuated the ability of DEX to suppress neuronal ERK/Syn1 activation, whereas knockdown of IL-1β enhanced the inhibitory effect of DEX (Figures 5D,E). These data identify microglia-derived IL-1β as a critical paracrine mediator linking inflammatory activation to neuronal ERK/Syn1 dysregulation in the coculture system.

We then used pharmacological modulators to position ERK within this pathway. Representative immunoblots showed that ERK inhibition with Trametinib reduced P-ERK/ERK and P-Syn1/Syn1, whereas ERK activation with EGF increased both readouts under inflammatory coculture conditions (Figure 5F). Quantification confirmed these effects (Figure 5G).

Together, these findings support a model in which microglia-derived IL-1β promotes neuronal ERK-dependent Syn1 phosphorylation and DEX interrupts this inflammatory signaling axis.

### 3.6 DEX suppresses microglial IL-1β through Acod1-dependent itaconate reprogramming under inflammatory priming

**FIGURE 6:**
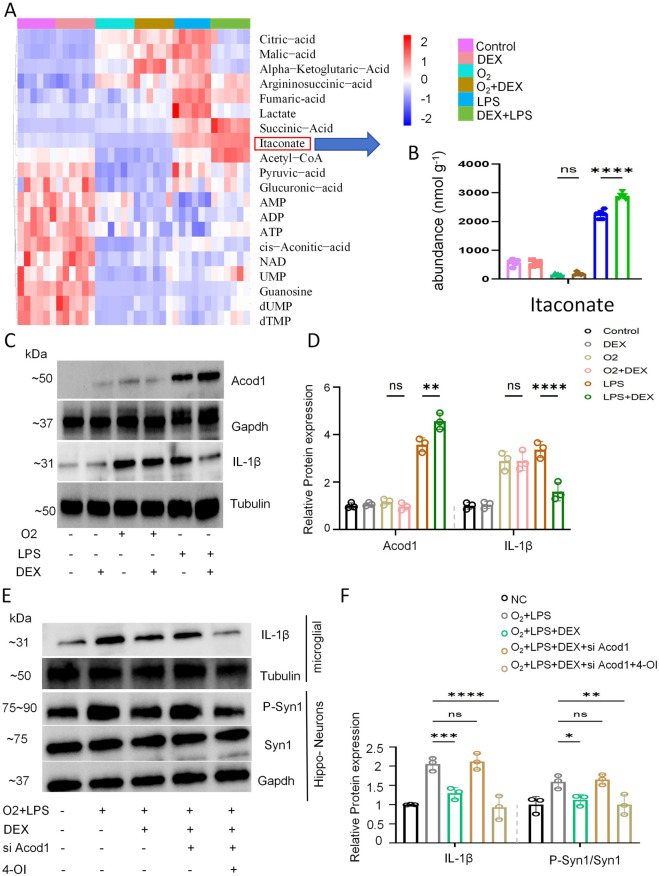
DEX suppresses microglial IL-1β through Acod1-dependent itaconate reprogramming under inflammatory priming. Primary microglial cell pellets were used for TCA cycle-related metabolite and itaconate profiling. Samples were randomized before injection, and QC evaluation was performed before group decoding. **(A)** Heat map of targeted metabolomic profiling of tricarboxylic acid (TCA) cycle-related metabolites in primary microglia exposed to normoxia, DEX alone, O_2_, O_2_+DEX, LPS, or LPS + DEX. The metabolomic profile indicates stimulus-dependent metabolic remodeling, with a prominent increase in itaconate under inflammatory priming. **(B)** Quantification of itaconate abundance in the indicated groups, showing that DEX significantly increased itaconate levels in LPS-stimulated microglia, but not under hyperoxic conditions alone (n = 6). **(C)** Representative immunoblots of Acod1 and IL-1β in primary microglia under O_2_- or LPS-based stimulation in the presence or absence of DEX. GAPDH and Tubulin were used as loading controls. **(D)** Densitometric quantification of Acod1 and IL-1β expression shown in **(C)**, demonstrating that DEX increased Acod1 expression and suppressed IL-1β preferentially in LPS-stimulated microglia (n = 3). **(E)** Representative immunoblots from the microglia-neuron coculture system used for functional validation of the Acod1-itaconate pathway. Knockdown of Acod1 attenuated the inhibitory effect of DEX on microglial IL-1β and neuronal P-Syn1/Syn1, whereas supplementation with 4-octyl-itaconate (4-OI) partially restored these effects. **(F)** Densitometric quantification of IL-1β in microglia and P-Syn1/Syn1 in hippocampal neurons shown in **(E)** (n = 3). Dashed vertical lines separate different targets; statistical comparisons were performed within each target, not between targets. Data are presented as mean ± SEM. For comparisons involving stimulation context and DEX treatment, two-way ANOVA followed by Sidak’s multiple-comparisons test was used. Acod1 knockdown and 4-OI rescue experiments were analyzed using one-way ANOVA followed by Tukey’s multiple-comparisons test. *P < 0.05, **P < 0.01, ***P < 0.001, ****P < 0.0001.

We next investigated the mechanism by which DEX suppresses microglial IL-1β. Targeted metabolomic profiling of TCA cycle-related metabolites in primary microglia revealed that DEX induced a stimulus-dependent metabolic response. The heat map showed that microglial metabolic remodeling differed substantially between hyperoxic and inflammatory conditions, with a particularly prominent signal involving itaconate under LPS stimulation (Figure 6A). Quantitative analysis confirmed that DEX significantly increased itaconate abundance in LPS-stimulated microglia but not under hyperoxic conditions alone (Figure 6B).

To determine whether this metabolic shift was associated with Acod1 activation, we examined Acod1 and IL-1β expression in microglia under O_2_- or LPS-based stimulation. Immunoblot analysis showed that DEX increased Acod1 expression and suppressed IL-1β preferentially in LPS-stimulated microglia (Figure 6C), and densitometric quantification confirmed this pattern (Figure 6D). These findings suggest that the anti-inflammatory effect of DEX is context dependent and is linked to Acod1-mediated itaconate production under inflammatory priming.

To test this hypothesis functionally, we perturbed the Acod1-itaconate pathway in the microglia-neuron coculture system. Knockdown of Acod1 attenuated the inhibitory effect of DEX on microglial IL-1β and neuronal P-Syn1/Syn1, whereas supplementation with 4-octyl-itaconate (4-OI) partially restored these effects (Figure 6E). Quantitative analysis confirmed the reversal and rescue effects on both microglial IL-1β and neuronal P-Syn1/Syn1 (Figure 6F).

Because the heat map in Figure 6A summarizes global metabolite remodeling, we further quantified individual TCA-related metabolites under LPS stimulation. Supplementary analysis showed that DEX altered multiple TCA-associated metabolites, including acetyl-CoA, citrate, itaconate, succinate, fumarate, malate, and α-ketoglutarate, consistent with itaconate-associated metabolic reprogramming in inflammatory microglia (Supplementary Figure S2).

Taken together, these results indicate that the anti-inflammatory action of DEX in microglia is stimulus dependent and is mediated, at least in part, through Acod1-dependent itaconate reprogramming under inflammatory priming, thereby limiting downstream neuronal Syn1 phosphorylation.

## 4 Discussion

In the present study, we identify a developmentally sensitive therapeutic window for DEX neuroprotection in a neonatal double-hit model of BPD-associated brain injury. Among the postnatal treatment regimens tested, DEX initiated at P3 produced the most consistent protective effects across lung pathology, survival, hippocampal injury, microglial reactivity, behavioral performance, and hippocampal functional connectivity. Taken together, our findings broaden the interpretation of DEX from a pulmonary intervention used in evolving BPD to a time-sensitive neuropharmacological modulator that suppresses microglia-driven inflammatory signaling in the developing brain. Mechanistically, the neuroprotective effects of DEX were associated with inhibition of microglia-derived IL-1β, attenuation of downstream neuronal ERK/Syn1 signaling, and engagement of Acod1-dependent itaconate reprogramming under inflammatory priming. These results support an immunometabolic framework for understanding the central actions of corticosteroids in neonatal injury and suggest that the efficacy of DEX may depend not only on dose and clinical indication, but also on developmental stage and inflammatory context.

An important implication of the present study is that the efficacy of DEX was strongly dependent on the timing of postnatal initiation. Although only three initiation time points were examined, the superior effects observed with the P3 regimen across structural, functional, and behavioral endpoints suggest that DEX responsiveness in this model is developmentally regulated rather than uniform across the early postnatal period. From a pharmacological perspective, this finding supports the concept that corticosteroid actions in the immature brain are shaped by a therapeutic window in which the balance between anti-inflammatory efficacy and developmental vulnerability may be optimized (Doyle et al., 2025). This interpretation is consistent with the notion that the neonatal brain undergoes rapid and stage-specific changes in synaptic maturation, glial reactivity, and circuit refinement, all of which may influence how glucocorticoid signaling is translated into biological outcome (Marengo et al., 2023). Our data therefore suggest that the neurological consequences of DEX exposure should not be viewed as fixed, but rather as context- and timing-dependent pharmacological effects.

The present findings also provide mechanistic support for a microglia-to-neuron signaling axis as a pharmacologically relevant target of DEX in neonatal brain injury (Borreca et al., 2024; Delahaye-Duriez et al., 2021; Jung et al., 2023). *In vivo*, double-hit injury was associated with increased hippocampal IL-1β, enhanced Syn1 phosphorylation, altered VGLUT1/VGAT-associated synaptic profiles, and glutamate/GABA imbalance, whereas DEX partially reversed these abnormalities. *In vitro* coculture experiments further indicated that microglia-derived IL-1β acted upstream of neuronal ERK/Syn1 activation, and that DEX interrupted this inflammatory cascade. These observations are important from a neuropharmacological standpoint because they link a clinically used corticosteroid to modulation of a defined inflammatory-synaptic pathway rather than to nonspecific suppression of tissue injury alone. Given the central role of ERK/Syn1 signaling in synaptic vesicle regulation and neurotransmitter release (Hsu et al., 2023; Lee et al., 2024; Zhou et al., 2023), the ability of DEX to restrain microglia-driven IL-1β signaling may represent a key mechanism by which inflammatory injury is translated into functional synaptic dysfunction in the developing hippocampus.

Another notable aspect of this study is the identification of Acod1-itaconate reprogramming as a mechanistically relevant component of DEX action under inflammatory priming. Recent work has highlighted that glucocorticoids can remodel cellular metabolism in parallel with their canonical transcriptional effects on inflammatory mediators (Stifel et al., 2022). In this context, our metabolomic and perturbation data suggest that the anti-inflammatory effects of DEX in microglia are not stimulus independent, but instead rely, at least in part, on a permissive inflammatory state that enables engagement of an Acod1-itaconate program. Functionally, disruption of this pathway attenuated the ability of DEX to suppress microglial IL-1β and downstream neuronal Syn1 phosphorylation, whereas restoration with 4-octyl-itaconate partially rescued these effects. These findings expand the pharmacological interpretation of DEX beyond conventional glucocorticoid signaling and support the view that immunometabolic state may be an important determinant of therapeutic responsiveness. Such a framework may be particularly relevant in inflammation-associated neonatal disorders, in which drug efficacy may depend not only on the administered agent itself, but also on the metabolic and inflammatory landscape of the target tissue (Auger et al., 2024; Wang et al., 2026).

The distinction between inflammatory priming and hyperoxic injury may be particularly important for interpreting the variable neurological effects of postnatal steroids (Bernis et al., 2022). Hyperoxia and prenatal inflammation are not interchangeable insults. Although both contribute to neonatal injury, they differ in their effects on cytokine induction, mitochondrial stress, and metabolic plasticity (Serdar et al., 2025). Our data suggest that prenatal inflammatory priming creates a microenvironment in which DEX can engage an Acod1-itaconate anti-inflammatory program, thereby suppressing IL-1β and limiting downstream neuronal signaling abnormalities. By contrast, hyperoxia alone may not provide the same immunometabolic context. This may help explain why hyperoxia-only models are insufficient to resolve the context-dependent central effects of corticosteroids and why clinically relevant inflammatory backgrounds should be incorporated into experimental models of BPD-associated brain injury (Jensen et al., 2023; Onland et al., 2023).

The present findings also refine the interpretation of lung-brain coupling in neonatal injury. Because DEX improved lung pathology and survival, indirect systemic or lung-brain axis-mediated contributions to the observed neuroprotection cannot be fully excluded (Parikh and Jain, 2026; Tréluyer et al., 2022). Nevertheless, the coculture and mechanistic experiments support the existence of a central component of DEX action that is superimposed on its pulmonary benefit. This distinction broadens the interpretation of corticosteroid response from a purely respiratory outcome toward a neuroimmune one.

Although these findings may have translational relevance, they should be interpreted cautiously. DEX is already used in preterm infants with evolving or severe BPD, yet its neurological consequences remain debated (Hay et al., 2023; Romijn et al., 2023). Our data suggest that corticosteroid response may depend not only on dose and timing but also on the inflammatory state of the neonate. More specifically, they raise the possibility that inflammation-associated BPD may represent a subgroup in which DEX has greater potential to confer central benefit. However, this possibility requires validation in human cohorts and should not be taken as a direct basis for clinical recommendation (Zhu et al., 2021).

Several limitations should be acknowledged. First, this study relied primarily on a rodent model and therefore cannot fully capture the complexity of human preterm brain injury or the heterogeneity of neonatal BPD. Second, although our data support a microglia-to-neuron signaling mechanism, cell-type specificity was inferred mainly from coculture experiments and tissue-level analyses rather than from cell-selective *in vivo* approaches. Third, downstream pathways linking Acod1-itaconate reprogramming to IL-1β suppression were not directly dissected in detail. Fourth, longer-term neurobehavioral outcomes beyond the juvenile period were not assessed. Finally, because only a limited number of treatment schedules were examined, the developmental specificity of the DEX therapeutic window remains only partially defined. Future studies should address these issues and further examine how developmental stage, inflammatory priming, and metabolic state interact to shape corticosteroid responsiveness in the injured immature brain.

In summary, the present study identifies a time-sensitive and mechanistically supported neuroprotective effect of DEX in a clinically relevant double-hit model of neonatal BPD-associated brain injury. Our findings suggest that DEX suppresses microglia-derived IL-1β-associated neuronal ERK/Syn1 signaling and that Acod1-dependent itaconate reprogramming contributes to this effect under inflammatory priming. These results expand the pharmacological interpretation of DEX beyond its established pulmonary use and support an immunometabolic framework for understanding corticosteroid actions in the developing brain. More broadly, they highlight the possibility that therapeutic efficacy in neonatal inflammatory brain injury may depend not only on the choice of drug, but also on the developmental and inflammatory context in which that drug is administered.

## Data Availability

The original contributions presented in the study are included in the article/Supplementary Material, further inquiries can be directed to the corresponding authors.
