# Supplementary material for: Developmentally sensitive neuropharmacological effects of dexamethasone in neonatal bronchopulmonary dysplasia-associated brain injury via microglial Acod1-itaconate/IL-1β signaling
Source: Front Pharmacol. 2026 Jul 8;17:1840628. doi: 10.3389/fphar.2026.1840628 (PMC13388472; doi:10.3389/fphar.2026.1840628)
Supplement: Supplementary file 1 [file Supplementaryfile1.docx]

**Supplementary materials**

**Table S1. Animal allocation, survival cohort, and sample use in downstream analyses**

| **Group** | **Survival cohort initial n** | **Male/Female in survival cohort** | **Cumulative deaths before P15** | **Cumulative deaths before P28** | **Alive/**  **censored at P28** | **Lung histology** | **Brain histology**  **/IF/TEM** | **WB/**  **ELISA** | **rs-fMRI** | **Behavior** | **Metabolomics** |
| --- | --- | --- | --- | --- | --- | --- | --- | --- | --- | --- | --- |
| Control | 40 | 20/20 | 0 | 0 | 40 | 6 | 6 | 4–6 | 10 | 10 | 7 |
| BPD | 40 | 20/20 | 12 | 22 | 18 | 6 | 6 | 4–6 | 10 | 10 | 7 |
| BPD+DEX-P1 | 40 | 20/20 | 12 | 16 | 24 | 6 | 6 | 4–6 | NP | NP | NP |
| BPD+DEX-P3 | 40 | 20/20 | 3 | 6 | 34 | 6 | 6 | 4–6 | 10 | 10 | 7 |
| BPD+DEX-P8 | 40 | 20/20 | 1 | 20 | 20 | 6 | 6 | 4–6 | NP | NP | NP |

Data are shown as the number of animals used in the independent survival cohort and predefined downstream analysis cohorts. The survival cohort was independent from animals used for tissue collection, rs-fMRI, behavioral testing, and metabolomic analysis. rs-fMRI and behavioral testing were performed in the same longitudinal functional cohort. Other tissue-based analyses were performed using predefined independent cohorts. Survival analysis included 40 pups per group. Deaths before P15 and P28 indicate cumulative spontaneous deaths within the survival cohort before the corresponding time points. Animals alive at P28 were censored at the end of follow-up. Planned euthanasia for tissue collection in separate cohorts was not counted as death. Lung histology was performed at P15 and P28. Brain histology, immunofluorescence, and TEM were performed using P15 hippocampal tissues. Terminal hippocampal metabolomic analysis was performed at P52. WB/ELISA indicates n = 4–6 biological replicates depending on the assay, with exact n values provided in the corresponding figure legends. NP, not performed. BPD+DEX-P1 and BPD+DEX-P8 were included in the DEX timing-screening experiment but were not used for downstream rs-fMRI, behavioral, or metabolomic analyses.

**Table S2. Antibodies used for western blotting, immunofluorescence, and immunocytochemistry**

| **Target** | **Application** | **Host** | **Vendor** | | **Catalog No.** | | **Dilution** | | **Normalization / Notes** | |
| --- | --- | --- | --- | --- | --- | --- | --- | --- | --- | --- |
| VGLUT1 | WB/IF | Rabbit | Proteintech | | 55491-1-AP | | WB 1:1000; IF 1:200 | | WB normalized to β-actin | |
| VGAT | WB/IF | Rabbit | Proteintech | | 14471-1-AP | | WB 1:1000; IF 1:200 | | WB normalized to β-actin | |
| Phospho-Synapsin I Ser9 | WB/IF | Rabbit | CST | | 67605 | | WB 1:1000; IF 1:200 | | WB normalized to total Synapsin I | |
| Synapsin I | WB | Rabbit | CST | | 5297S | | WB 1:1000 | | Normalized to β-actin | |
| IL-1β | WB | Rabbit | HUABIO | | ET1701-39 | | WB 1:1000 | | Normalized to Tubulin | |
| Acod1/Irg1 | WB | Rabbit | Abcam | | ab222411 | | WB 1:1000 | | Normalized to GAPDH or Tubulin | |
| Phospho-ERK1/2 | WB | Rabbit | HUABIO | | SC58-01 | | WB 1:1000 | | Normalized to total ERK1/2 | |
| ERK1/2 | WB | Rabbit | HUABIO | | SA43-03 | | WB 1:1000 | | Normalized to β-actin | |
| Flk-1/VEGFR2 | WB | Rabbit | CST | | 2463S | | WB 1:1000 | | Normalized to β-actin | |
| β-actin | WB | Mouse | Proteintech | | 66009-1 | | WB 1:5000 | | Loading control | |
| α-Tubulin | WB | Rabbit | Proteintech | | 11224-1-AP | | WB 1:5000 | | Loading control | |
| Iba1 | IF/ICC | Rabbit | CST | | 17198 | | IF/ICC 1:500 | | Microglia marker | |
| MAP2 | ICC | Rabbit | Millipore | | AB5622 | | ICC 1:500 | | Neuronal marker | |
| **Secondary antibody** | | **Application** | | **Host** | | **Vendor** | | **Catalog No.** | | **Dilution** |
| HRP-conjugated anti-mouse IgG | | WB | | Goat | | Proteintech | | SA00001-1 | | 1:5000 |
| HRP-conjugated anti-rabbit IgG | | WB | | Goat | | Proteintech | | RGAR301 | | 1:5000 |
| Alexa Fluor 594-conjugated anti-rabbit IgG | | IF/ICC | | Goat | | Proteintech | | SA00013-4 | | 1:500 |
| Alexa Fluor 488-conjugated anti-rabbit IgG | | IF | | Goat | | Proteintech | | SA00013-2 | | 1:500 |
| Alexa Fluor 647-conjugated anti-rabbit IgG | | IF | | Goat | | Proteintech | | SA00014-9 | | 1:500 |
| DAPI | | Nuclear stain | | — | | Beyotime | | P0131 | | Nuclear counterstain |

WB, western blotting; IF, tissue immunofluorescence; ICC, immunocytochemistry. For western blotting, phosphorylated proteins were normalized to their corresponding total proteins, whereas total proteins were normalized to β-actin, Tubulin, or GAPDH as indicated. For immunofluorescence and immunocytochemistry, species-appropriate Alexa Fluor-conjugated secondary antibodies were used. All PVDF membranes were blocked using rapid protein blocking buffer for western blotting (Beyotime Biotechnology, China).


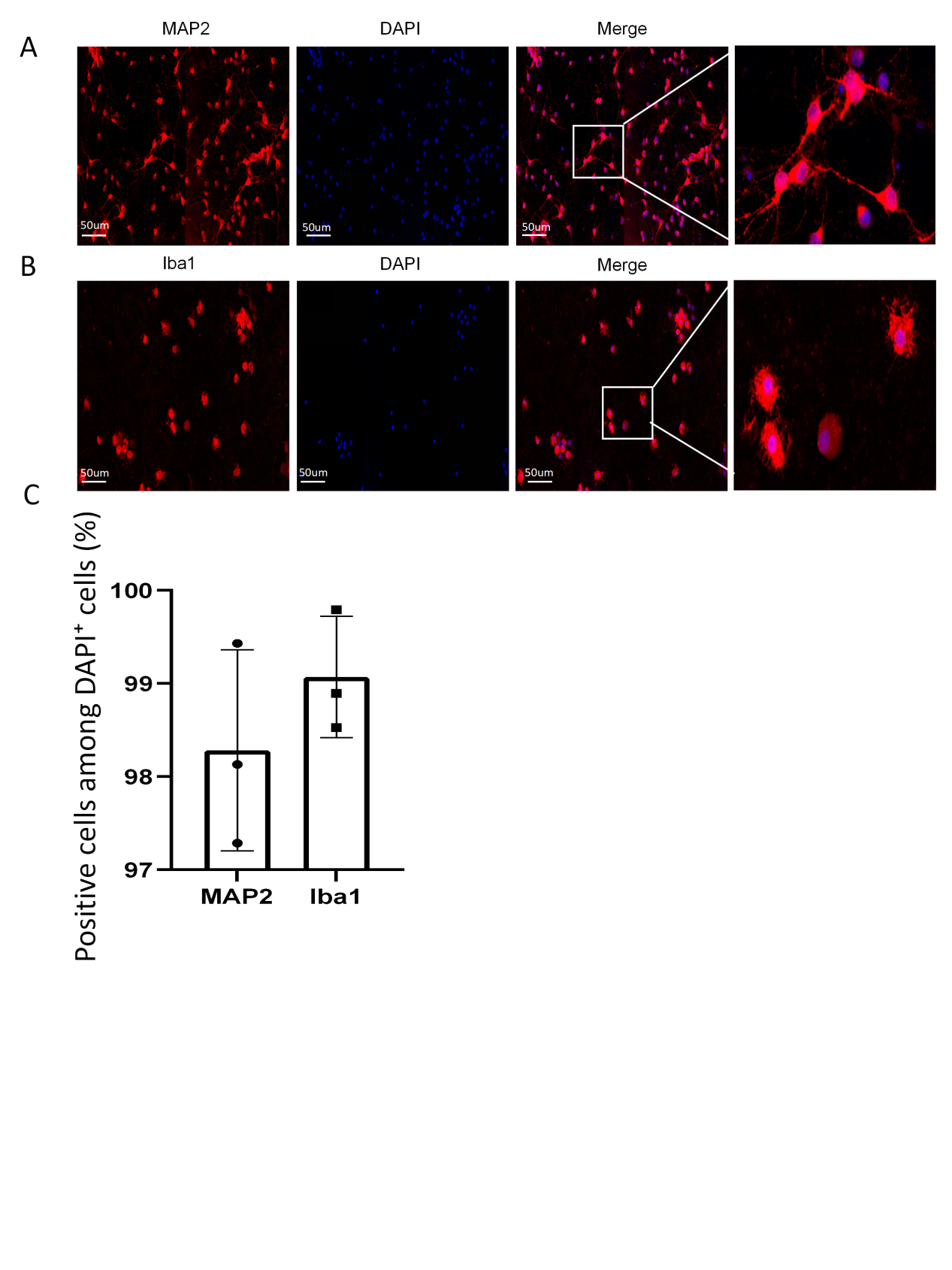


**Figure S1.** Identification and purity assessment of primary hippocampal neurons and primary microglia. (A) Representative immunocytochemistry images of primary hippocampal neurons stained for MAP2. Nuclei were counterstained with DAPI. The boxed region is shown at higher magnification on the right. (B) Representative immunocytochemistry images of primary microglia stained for Iba1. Nuclei were counterstained with DAPI. The boxed region is shown at higher magnification on the right. (C) Quantification of cell purity. Neuronal purity was calculated as the percentage of MAP2⁺ cells among DAPI⁺ cells, and microglial purity was calculated as the percentage of Iba1⁺ cells among DAPI⁺ cells (n = 3). Both primary hippocampal neuron and microglial cultures showed purity greater than 95%. Data are presented as mean ± SEM. Scale bars, 50 μm.

**
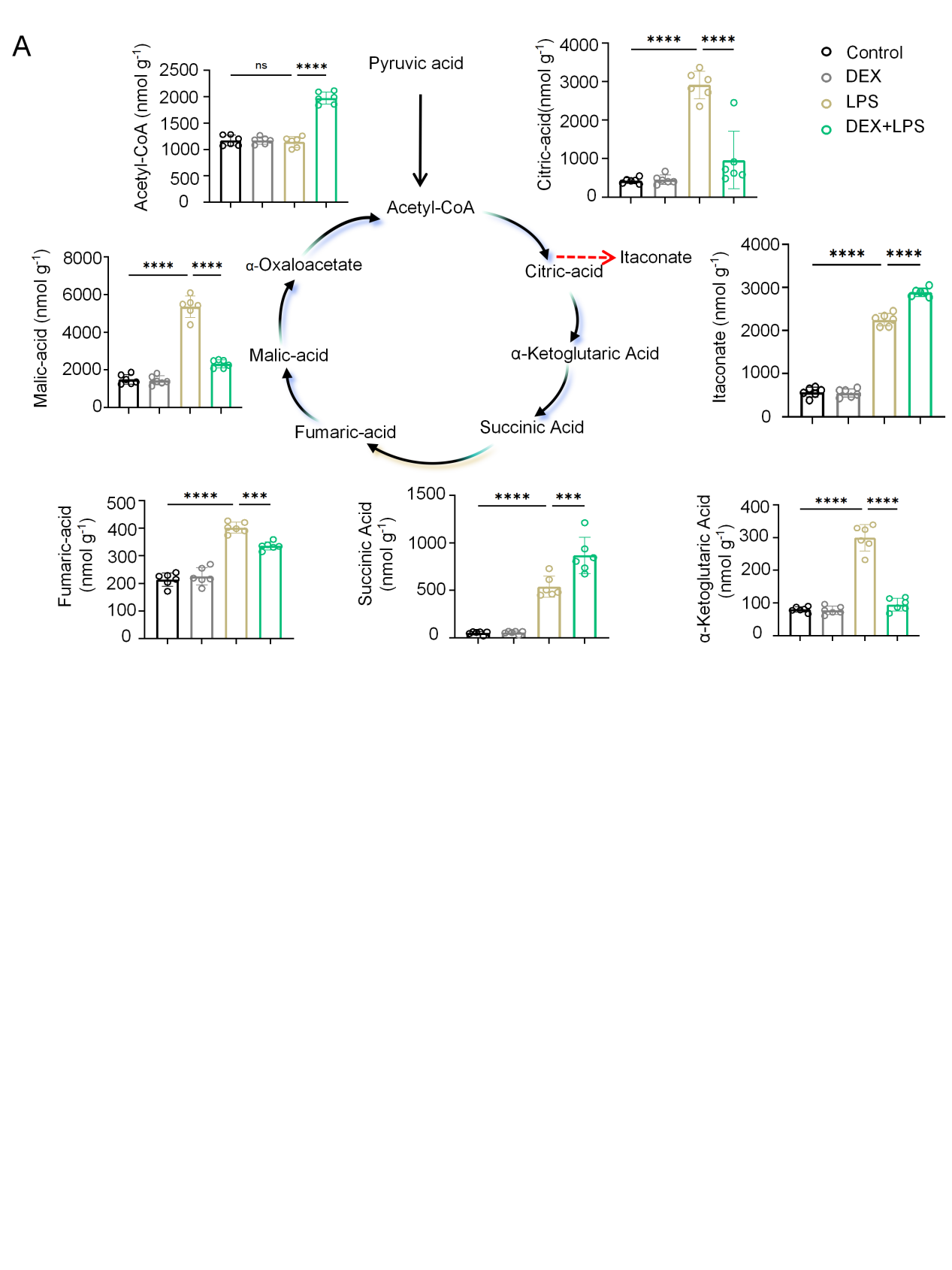
**

**Figure S2. Quantification of individual TCA-related metabolites reveals DEX-induced itaconate-associated reprogramming in LPS-stimulated microglia.** Primary microglia were stimulated with LPS in the presence or absence of DEX, and intracellular metabolites were quantified by targeted LC-MS/MS. Bar plots show the relative abundance of indicated TCA-related metabolites, including acetyl-CoA, citrate, itaconate, succinate, fumarate, malate, and α-ketoglutarate. DEX treatment increased itaconate-associated metabolic remodeling in LPS-stimulated microglia, consistent with the global metabolic pattern shown in Fig. 6A. Data are presented as mean ± SEM. Data were analyzed using two-way ANOVA followed by Sidak’s multiple-comparisons test to assess the main effects of LPS, DEX, and their interaction. Significance bars indicate the post hoc comparisons shown in the graph. **P* < 0.05, ***P* < 0.01, ****P* < 0.001, *****P* < 0.0001; ns, not significant.
